# Burden of Chronic Heart Failure in Romania

**DOI:** 10.3390/healthcare10010107

**Published:** 2022-01-06

**Authors:** László Lorenzovici, Andrea Bârzan-Székely, Szabolcs Farkas-Ráduly, Bogdan C. Pană, Marcell Csanádi, Nona Delia Chiriac, Zoltán Kaló

**Affiliations:** 1 Syreon Research Romania, 540004 Tirgu Mures, Romania; lorenzovici@syreon.ro (L.L.); farkas_raduly_szabolcs@syreon.ro (S.F.-R.); 2 Department of Doctoral Studies, George Emil Palade University of Medicine, Pharmacy, Science, and Technology of Târgu Mureş, 540142 Tirgu Mures, Romania; 3 Department of Public Health, University of Medicine and Pharmacy “Carol Davila” Bucharest, 020021 Bucharest, Romania; bogdan.pana@umfcd.ro; 4 Syreon Research Institute, 1142 Budapest, Hungary; marcell.csanadi@syreon.eu (M.C.); kalo.zoltan@semmelweis-univ.hu (Z.K.); 5 Novartis Pharma Services, 020334 Bucharest, Romania; nona.chiriac@novartis.com; 6 Center for Health Technology Assessment, Semmelweis University, 1091 Budapest, Hungary

**Keywords:** chronic heart failure, CHF, burden of disease, cost of hospitalization, Romania

## Abstract

Chronic heart failure (CHF) affects millions of people across the world, with increasing trends in prevalence, putting ever increasing pressure on the healthcare system. The aim of this study was to assess the financial burden of CHF hospital care on the public healthcare sector in Romania by estimating the number of inpatient episodes and the associated costs. Additionally, societal costs associated with missed work and premature death of CHF patients were also estimated. The national claims database was analyzed to estimate the number of CHF patients. Cost data was extracted from a pool of nine public hospitals in Romania. In 2019, 375,037 CHF patient episodes were identified on specific wards at the national level. The average cost calculated for the selected nine hospitals was EUR 996. The calculated weighted national average cost per patient episode was EUR 1002, resulting in a total cost of EUR 376 million at the national level. The cost of workdays missed summed up to EUR 122 million, while the annual costs associated with the premature death of CHF patients was EUR 230 million. In conclusion, the prevalence of CHF in Romania is high, accounting for a large proportion of hospitalizations, which translates into large costs for the national payer.

## 1. Introduction

Chronic heart failure (CHF), having a current prevalence of over 64.3 million people in the world [1], with increasing trends [2], is considered one of the most common diseases [3]. The most common symptoms include fatigue, swelling of the limbs, weakness, and shortness of breath [4]. The prevalence varies across geographical regions, reported values being of 1.3% in Australia [5], between 1.3% and 6.7% in Asia [6], and between 1 and 2% in Europe [7], with five of the six countries with the highest age-standardized prevalence rates of HF being located in Central-Eastern Europe [8]. Studies show a 50% mortality rate of CHF patients within 5 years of diagnosis [9,10].

CHF is responsible for 1–5% of all hospital admissions [11,12]. The high prevalence of the illness and the associated costs account for a substantial economic burden on the healthcare systems. Studies show costs associated with the hospitalization of CHF patients of USD 24,383 per patient in the US [13], USD 10,123 in Canada [14], EUR 2146 in Spain [15], USD 639 in Poland [16], USD 2285 in Sweden [17], EUR 5900 in Italy [18], and EUR 2854 in Ireland [19]. Reviews show that the proportion of inpatient costs of the total costs vary from one country to another and is between 47% and 96% [20].

Cost measurement is necessary, especially in the case of countries using multiple payment mechanisms besides performance-based reimbursement for inpatient care, such as the DRG system currently employed in Romania, which is meant to reimburse the average cost of a given illness, or if the performance payment is not managed properly. Currently in Romania, inpatient care is financed not only through the DRG system, but also through additional mechanisms, such as salary subsidies, which, in many cases, surpass the value of the DRG reimbursement [21]. Despite this “additional” financing, numerous hospitals receive funds from local authorities (or other public payers) to cover their costs. The lack of proper funding leads to financial deficit, translating into a lack of medical supplies and medication in those hospitals where the public payer does not have the financial possibility to cover the gap, leading to inequalities in access to medical services.

To explore whether the national reimbursement level of CHF care is appropriate with respect to the cost of treatment, a real-world cost measurement should be performed. The results of such analysis could be used to propose modifications in the reimbursement system to cover all actual treatment costs from the perspective of hospitals.

From a global burden of disease standpoint, in 2019 ischemic heart disease (IHD) was one of the top-ranked causes of DALYs in both the 50–74-year and 75-years-and-older age groups [22]. CHF produced disability is strongly linked to IHD and hypertensive heart diseases, which represent, 14.56% and 2.46% of total DALYs in Romania [23].

The main aim of our study was to estimate the financial burden of CHF hospital care on the public healthcare sector in Romania. First, we aimed to define the number of inpatient episodes, then estimate the associated costs. Finally, we also attempted to take into account the costs outside hospitals, such as the societal costs associated with missed work and the premature death of CHF patients.

## 2. Materials and Methods

### 2.1. Data Sources

In order to assess the volume of care for CHF patient episodes in Romanian public hospitals, anonymized digital records were used, containing the data of discharged patients reported to the National Health Insurance House (NHIH) for reimbursement purposes for the year 2019. These included the following parameters: principal and secondary diagnoses, discharging hospital, discharging department, age, sex, environment of residence, DRG, length of stay (LoS), and intensive care unit (ICU) LoS.

The cost assessment was based on detailed economic data from a selected pool of public Romanian hospitals. These were selected according to their hospital category (clinical, county, and city hospitals), their geographic location, to ensure the pool’s representativity for the Romanian public healthcare system, and access to individual level cost data. Anonymized medical patient parameters were collected from the hospitals’ databases, which have been prepared to be sent to the NHIH for reimbursement purposes. Cost data and service utilization was accessed through the hospital controlling system implemented in the selected hospitals. Calculations performed in these data resulted in the average cost per patient episode per each hospital category. The calculations were extrapolated to estimate the national level costs according to the number of patient episodes discharged in 2019 in Romania.

After considering the criteria for hospital selection (hospital category, geographical location, access to cost data), nine public hospitals were selected, three of which were clinical hospitals, three county hospitals, and three city hospitals. From a geographical point of view, four of these hospitals can be found in Macroregion 1 of the country, two in Macroregion 2, one in Macroregion 3, and two in Macroregion 4, with at least one hospital having been selected from each macroregion.

The calculation of costs associated with missed work and the premature death of CHF patients was based on national level information regarding average gross salary, based on data reported by the National Institute of Statistics of Romania. To assess the societal cost of the care of CHF patients, the average length of stay was calculated for patients up to 64 years; expert opinion (a group of family doctors/general practitioners) was used to estimate the length of the medical leave. This study does not take into account early retirement or a lengthier medical leave. Societal costs also include losses resulting from premature death for the active population [24,25,26]. The total number of years lost has been calculated by subtracting the ages of deceased patients from 65 years (the average age of retirement) and summing the active-life years lost.

### 2.2. Study Population and Inclusion Criteria

The following inclusion criteria were defined: patients with at least a principal or secondary diagnosis code (according to ICD-10 AM) of CHF based on the discharge information, including I50.0 (Congestive heart failure), I50.1 (Left ventricular failure), I50.9 (Heart failure, unspecified), or I11.0 (Hypertensive heart disease with (congestive) heart failure); minimum age of 18 years.

For the cost assessment, patients with the above-mentioned diagnosis codes were selected from the following departments: Cardiology, Cardiovascular surgery, Chronic care, Geriatrics, Internal Medicine, Cardiac Rehabilitation, and Coronary Intensive Care Unit (CICU). Even though CHF diagnoses can also be found at patient episodes discharged by other departments, in these situations we considered that the hospitalization costs were mainly determined by the other diseases/surgical intervention, e.g., cholecystectomy.

### 2.3. Data Analyses

Romanian hospitals are not required by law to record resource use and unit costs for most inputs at the patient level (except for drugs and medication) [27], but the selected hospitals have implemented a cost controlling system, with the ability to provide good quality cost data. The cost measurement method used for this study was based on the controlling [28] data and assessed the costs with a “top-down” approach. Accordingly, the average cost per service was defined by dividing the total cost with the volume of services performed [27]. The study includes direct costs of care (labor costs of the medical and non-medical staff, drugs and medication, medical and non-medical supplies, spare parts, utility costs) and internal services (diagnostics, transfusion, ICU stay, sterilization, etc.) and the overhead-type hospital costs of care (including administrative costs, maintenance, HR (human resources), accounting, safety and security, IT, statistics, and other hospital category costs that cannot be attributed at patient level [21]). Amortization and depreciation were not included in the calculations. All cost data have been recorded in Romanian Leu (RON), and for the conversion the yearly average exchange rate was used, as calculated by the Romanian National Bank for 2019 (1 EUR = 4.8371 RON). Inpatient care (a patient hospitalization episode) was considered to start at the moment of admission and end at the time of discharge. This time span was considered a hospital episode, regardless of whether or not the patient had previous or subsequent hospitalizations.

All costing calculations were based on the actual hospital spending, irrespective of the reimbursement or fee received by the hospitals per patient episode.

The estimated error margins were 1.96, and 95% confidence intervals were calculated for the cost data. Additionally, the minimal number of patient episodes required for the representativity of costs measurement was calculated using Cochran’s formula.

The weighted national level average cost per patient episode calculations was based on the average cost per patient episode per hospital category, weighted by the number of CHF patient episodes discharged from all hospitals of the given category, because the various hospital categories discharge a different number of patients.

## 3. Results

### 3.1. National Level Patient Population

According to the 2019 data, there have been 590,730 CHF patient episodes reported to the NHIH for reimbursement purposes, of which 375,037 CHF patient episodes met our inclusion criteria from the studied departments. Importantly, this does not represent the number of CHF patients in Romania, but the number of patient episodes. The majority of these patient episodes were treated at the Cardiology (28.8%) and Internal Medicine (28.2%) departments, most patient episodes being reported from clinical hospitals.

### 3.2. Cost Data

In the nine selected public hospitals, 20,296 CHF patient episodes were treated in 2019 (representing 5.4% of all CHF patient episodes reported on a national level from the selected departments and 3.4% of all CHF patient episodes on a national level from all departments). According to Cochran’s formula, based on the heterogeneity of the cost data, the study population exceeded the minimum number of patient episodes needed for every hospital category, in order to be statistically representative.

The patient parameters of the study population can be found in Table 1.

The average length of stay (ALoS) and average length of stay at the ICU (ALoS ICU) are two of the key cost drivers at the patient level. The reported national-level CHF ALoS was pf 6.7 days, regardless of the hospitalization cause (5.2% lower compared to the parameters of the study population). ICU ALoS was calculated for all CHF patient episodes meeting the inclusion criteria, including those not treated at the ICU.

The cost analysis was stratified according to the category of the hospitals, as can be seen in Table 2.

The highest costs were reported from clinical hospitals, having the highest professional level and treating the more severe and complex cases.

CHF patients have mostly non-surgical health issues (“medical” cases), so the costs associated with surgical services (e.g., operating room costs) were very low. For them, the costs associated with their stay on the medical ward were the highest cost component, as shown in Table 3. As many patients were admitted to the hospital through the emergency care unit, these costs were also high, as were the ICU costs, as some patients received treatment at the ICU (5.6% ICU patient episodes of all studied CHF patient episodes), and the costs associated were substantial.

### 3.3. Economic Burden of Inpatient Care of CHF Patients

Based on the calculated average cost per inpatient episode per hospital level (Table 2) and the number of patient episodes on a national level per hospital level, the weighted average cost per patient episode was calculated, resulting in EUR 1002.05. Because, in 2019, there had been 375,037 patient episodes reported to the NHIH for reimbursement purposes, the total budget impact on the public payer’s budget was calculated to be almost EUR 376 million, representing 4.35% of the NHIH budget (including reimbursement for health services, drugs and medication, and national health programs).

### 3.4. Societal Costs Associated with CHF Patients

The average length of stay was calculated for patients up to 64 years, being 6.4 days. This number was rounded up to 7, and 2 days were deducted for the weekends. According to expert opinion (a group of family doctors/general practitioners), CHF patients are granted 10 days of medical leave, again, deducting 2 days for the weekend. The resulting 13 days (5 + 8) of missed work were multiplied by the gross average wage for 2019 for Romania, including insurance contribution for work, to calculate the societal loss. The average gross monthly wage was EUR 1067 (according to the National Institute of Statistics of Romania). Calculating for 13 days, this results in EUR 694, along with social contributions of EUR 6, thus making the total cost of wages lost per patient episode EUR 700. In 2019, there had been 174,074 patient episodes for patients under 65 years. All this sums up to EUR 122 million.

After subtracting the ages of deceased patients from 65 years, the total number of life years lost was 17,519 for CHF patients treated at the selected hospital departments. This, multiplied by the average monthly gross wage and the social contributions (multiplied by 12, for each month), results in EUR 230 million.

## 4. Discussion

This study provides updated epidemiological and economic data on CHF in Romania. Results show a total financial burden on the healthcare system of EUR 376 million and an additional societal cost of EUR 122 million, associated with days of work missed, along with another EUR 230 million associated with the premature death of CHF patients.

Our results regarding the volume of the hospital care observed are in line with previous studies [29]; however, when put in an international setting [30], it becomes clear that the average age of hospitalized CHF patients was lower than in the case of other countries, which could be the result of the cumulated presence of risk factors.

These findings are consistent with the results of previous studies in Romania [31] in terms of costs and medical parameters. The differences in results can be explained through the increase in wages and labor costs incurred during the past few years in the Romanian public healthcare sector and the diagnosis codes included.

Cost variation between different hospital levels has been observed. One possible explanation for this difference could be the difference in severity levels of the patients, but this cannot be objectively evaluated by the Romanian DRG or coding system.

Our study has several strengths. First, the volume of care assessment was based on the entire patient population reported by public and private hospitals to the NHIH for reimbursement, both in acute and chronic care. However, this also carries some limitations, as a small proportion of the patients might not be included in these reports if the healthcare unit they were treated at does not report to the NHIH. The chances of this phenomenon are considered to be very low for this pathology.

The rehabilitation services in the Romanian healthcare system are underdeveloped, representing significant unmet needs [32]. If services were offered to all patients requiring rehabilitation, the burden of disease would become much higher for the public payer. Additionally, the study does not comprise one-day hospitalizations and ambulatory care; thus, the total burden for the healthcare system is rather underestimated.

Another limitation of the study was that it includes all the patient episodes with a principal or secondary diagnosis of CHF. However, it is possible for the patients to have been admitted for a different ailment, their status as a CHF patient not requiring immediate care, which could result in an overestimation of the total burden of inpatient care. On the other hand, the patients admitted to hospital departments other than the ones included in the study, because of their CHF diagnosis, could also have other comorbidities, which would increase the total burden of the disease. As a result, this study underestimates the total burden.

Cost per patient episode has been calculated based on the data from a pool of nine public Romanian hospitals, accounting for 5.4% of all reported CHF patient episodes in 2019 from the selected departments; thus, extrapolation at the national level may not be precise. However, because of the random selection of hospitals, and the uniform wage system used in Romania, their results are considered highly representative of the Romanian public hospital system.

One of the strengths of the study is the fact that the calculations also consider the societal costs associated with the treatment of CHF patients. However, these only consider the sick leave granted by general practitioners or family doctors, but it is also possible for specialist doctors to grant additional days of sick leave. However, this practice is not very common. Nonetheless, not considering these, the study underestimates the total societal cost. In order to assess the total global burden of disease, other indicators should also be considered, such as DALYs, but exactly quantifying the economic burden is not possible based on the available data.

Although it is fairly comprehensive, the authors mention as a caveat, the possibility that some patient episodes are not captured in the study. Our results specifically address the issue of CHF patients treated in the inpatient setting. It is important to stress this aspect because different management forms have different associated costs.

## 5. Conclusions

Based on the data gathered for 20,296 CHF patient episodes, the average cost per patient episode is 996 EUR, but the weighted average cost on a national level (weighing with the number of patient episodes discharged per hospital category) results in EUR 1002.05. The highest costs are attributed to the highest category hospitals (clinical hospitals), and hospital ward stay is responsible for the highest share of the costs (as CHF cases are highly medical, few of them being surgical). Taking into account all patient episodes reimbursed by the NHIH, the total budget impact sums up to EUR 376 million per year.

Based on the length of hospitalization and the average gross monthly salary, the societal loss because of reduced work capacity of CHF patients sums up to about EUR 122 million per year, while the societal loss generated by the premature yearly mortality of CHF patients amounts to EUR 230 million.

## Figures and Tables

**Table 1:** CHF patient episode parameters.

Factor	Male (95% CI)	Female (95% CI)	Total (95% CI)
	(*n* = 9533)	(*n* = 10,763)	(*n* = 20,296)
Age (years)	68.2 (67.8 to 68.6)	71.9 (71.5 to 72.2)	70.2 (69.8 to 70.5)
ALoS (days)	7.0 (6.8 to 7.2)	7.1 (7.0 to 7.3)	7.1 (7.0 to 7.2)
ALoS (ICU) (days) all patient episodes	0.31 (0.22 to 0.39)	0.28 (0.21 to 0.36)	0.29 (0.21 to 0.38)
ALoS (ICU) (days) ICU patient episodes	4.9 (4.6 to 5.2)	4.95 (4.7 to 5.2)	4.9 (4.6 to 5.2)

**Table 2:** Average cost (EUR) of CHF patient episodes stratified by hospital level.

Hospital Level	Average Cost/Episode	No. of Studied Patient Episodes	No. of National Level Patient Episodes
Clinical	1308	7083	143,107
County	852	9216	114,929
City	776	3997	117,001
Total	996	20,296	375,037

**Table 3:** Cost structure (EUR) of CHF inpatient treatment.

Factor	Value (95% CI)
Average total cost/patient episode	995.9 (927.2 to 1064.7)
Hospital ward costs	499.5 (484.1 to 514.9)
ICU costs	143.9 (90.3 to 197.5)
Medication costs	49.2 (44.9 to 53.6)
Operating room costs	0.49 (0.01 to 0.96)
Emergency care services costs	159.7 (148.8 to 170.6)
Diagnostic services costs	64.1 (61.1 to 67.0)
Other costs	26.3 (24.4 to 29.3)
Administrative costs	52.3 (48.2 to 56.4)

## Data Availability

The data presented in this study are available on request from the corresponding author.
